# Mechanisms Underlying Vascular Inflammaging: Current Insights and Potential Treatment Approaches

**DOI:** 10.14336/AD.2024.0922

**Published:** 2025-10-18

**Authors:** Ying Zeng, Francesco Buonfiglio, Jingyan Li, Norbert Pfeiffer, Adrian Gericke

**Affiliations:** Department of Ophthalmology, University Medical Center of the Johannes Gutenberg-University Mainz, Langenbeckstr.1, 55131 Mainz, Germany

**Keywords:** inflammaging, vasculature, age-related vascular disorders, pathophysiology, treatment, approaches

## Abstract

Inflammaging refers to chronic, low-grade inflammation that becomes more common with age and plays a central role in the pathophysiology of various vascular diseases. Key inflammatory mediators involved in inflammaging contribute to endothelial dysfunction and accelerate the progression of atherosclerosis. In addition, specific pathological mechanisms and the role of inflammasomes have emerged as critical drivers of immune responses within the vasculature. A comprehensive understanding of these processes may lead to innovative treatment strategies that could significantly improve the management of age-related vascular diseases. Emerging therapeutic approaches, including cytokine inhibitors, senolytics, and specialized pro-resolving mediators, aim to counteract inflammaging and restore vascular health. This review seeks to provide an in-depth exploration of the molecular pathways underlying vascular inflammaging and highlight potential therapeutic interventions.

## 1. Introduction

Inflammaging refers to a chronic, low-grade inflammatory state that intensifies with aging [1, 2]. Unlike acute inflammation, which is a temporary and protective response, inflammaging is a persistent condition that escalates during senescence, contributing to the development of various age-related diseases [3]. In the vasculature, inflammaging is characterized by elevated levels of systemic inflammatory markers, such as C-reactive protein (CRP), interleukin-6 (IL-6), and tumor necrosis factor-alpha (TNF-α) [4]. These key immune mediators indicate ongoing inflammatory processes that impair endothelial function, increase oxidative stress, and reduce nitric oxide (NO) bioavailability [5]. Endothelial cells, which are essential for maintaining vascular health, can become dysfunctional under chronic inflammation, leading to impaired vasodilation, increased arterial stiffness, and a pro-thrombotic state [6]. The strong connection between inflammaging and vascular dysfunction plays a major role in the development of age-related vascular diseases [7]. For example, these pathogenic processes promote the formation and destabilization of atherosclerotic plaques, raising the risk of cardiovascular events such as coronary artery disease and stroke [8]. Additionally, chronic inflammation associated with aging contributes to increased arterial stiffness and hypertension, further aggravating vascular damage [9]. Inflammaging also plays a role in the pathogenesis of vascular dementia, as persistent inflammation can compromise the blood-brain barrier (BBB), promoting chronic neuroinflammation [10, 11]. Recent research has highlighted the involvement of vascular inflammaging in ocular diseases with prominent vascular mechanisms, such as age-related macular degeneration (AMD) and diabetic retinopathy (DR) [12, 13].

The primary goal of this review is to provide an updated overview of the current understanding of inflammaging in vascular tissues, and to present potential therapeutic strategies aimed at mitigating its harmful effects and improving vascular health in the aging population.

## 2. Inflammaging: General Concepts

### 2.1 Inflammatory Markers

Research consistently shows that elderly individuals exhibit elevated inflammatory markers compared to younger populations, even in the absence of overt disease [14]. For example, the Baltimore Longitudinal Study of Aging found significantly higher levels of CRP and IL-6 in older adults, which were associated with increased arterial stiffness and endothelial dysfunction [15]. These findings highlight the pervasive impact of inflammaging on vascular health and suggest that targeting inflammation may help manage age-related vascular diseases [5]. Moreover, epidemiological studies have firmly linked inflammaging to vascular dysfunction, showing a significant correlation between age-related increases in systemic inflammation and the prevalence of vascular disorders [16]. Large-scale cohort studies, such as the Framingham Heart Study and the Cardiovascular Health Study, have reported that levels of inflammatory markers like CRP and IL-6 tend to rise with age and are predictive of cardiovascular events and mortality [17, 18]. Individuals with higher baseline CRP levels have a markedly increased risk of coronary heart disease [19, 20]. Similarly, the Cardiovascular Health Study found that elevated IL-6 levels were associated with a greater incidence of coronary heart disease and heart failure in older adults [18, 21, 22].

In addition to CRP and IL-6, other biomarkers have gained attention for their role in vascular inflammaging. Soluble tumor necrosis factor receptor 1 (sTNFR1), a marker of TNF-α signaling, has emerged as a strong predictor of both vascular aging and cardiovascular events. Elevated levels of sTNFR1 have been linked to worse vascular outcomes, suggesting its potential as a therapeutic target [23]. Growth and differentiation factor 15 (GDF-15), a stress response cytokine that increases with age, is another promising biomarker, as it correlates with vascular inflammation and dysfunction. Elevated GDF-15 levels have been associated with poor cardiovascular outcomes, making it useful for both diagnosis and treatment [24]. Additionally, soluble urokinase plasminogen activator receptor (suPAR) has gained recognition as a biomarker for chronic inflammation and endothelial dysfunction. Higher suPAR levels have been linked to increased arterial stiffness, atherosclerosis progression, and higher mortality rates, particularly in older populations [25]. These findings underscore the value of integrating newly identified biomarkers into the broader assessment of vascular inflammaging.

### 2.2 Genetic Factors

Genetic factors play a pivotal role in modulating the inflammatory response associated with aging, influencing individual susceptibility to inflammaging and its vascular consequences [26]. Polymorphisms in genes encoding pro-inflammatory cytokines and other inflammatory mediators contribute to variations in baseline inflammatory levels and the risk of developing vascular diseases [27]. For example, the -174G/C and -572G/C polymorphisms in the IL-6 gene have been linked to elevated circulating IL-6 levels and an increased risk of cardiovascular events [28, 29]. Similarly, variations in the promoter region of the TNF-α gene can influence TNF-α production, affecting the risk of atherosclerosis and other inflammatory conditions [30]. Epigenetic modifications are also believed to play a role in inflammaging. Age-related changes in DNA methylation patterns, for example, can upregulate pro-inflammatory genes, exacerbating the impact of inflammaging on vascular health [31, 32]. Additionally, genome-wide association studies have identified numerous loci associated with inflammatory biomarkers and vascular disease risk, further illustrating the complex genetic architecture underlying inflammaging [33]. Understanding the genetic and epigenetic factors contributing to inflammaging is essential for developing personalized therapeutic strategies. Identifying individuals with a genetic predisposition to heightened inflammatory responses could enable targeted interventions aimed at mitigating the effects of inflammaging on vascular health.

## 3. Insights into the Molecular Pathways of Vascular Inflammaging

### 3.1 Role of Oxidative Stress in Aging Processes

Oxidative stress occurs when there is an imbalance between the production of reactive oxygen species (ROS) and reactive nitrogen species (RNS) and the body’s endogenous antioxidant defenses, leading to the accumulation of ROS and RNS, which may cause significant cellular damage [34]. These species are key mediators of inflammaging in the vasculature, contributing to the aging process and the development of various vascular diseases, such as atherosclerosis and hypertension [35]. In vascular cells, excess ROS primarily originate from mitochondrial oxidative metabolism and the activity of enzymes like NADPH oxidase (NOX), xanthine oxidase, and uncoupled endothelial nitric oxide synthase (eNOS) [36, 37]. ROS, including superoxide anions (O_2_^•-^), hydrogen peroxide (H_2_O_2_), and hydroxyl radicals (·OH), initiate lipid peroxidation, protein oxidation, and DNA damage, compromising cellular structure and function [38]. Polyunsaturated fatty acids in cell membranes are particularly vulnerable to oxidative damage by ROS, especially ·OH, leading to the formation of lipid peroxides and reactive aldehydes like malondialdehyde (MDA) and 4-hydroxynonenal (4-HNE). These byproducts disrupt membrane integrity, altering cellular signaling and function. Moreover, lipid peroxidation products can act as secondary messengers, amplifying inflammation by activating nuclear factor kappa-light-chain-enhancer of activated B cells (NF-κB), creating a vicious cycle of oxidative stress and chronic inflammation within vascular tissues [39]. Proteins are also targeted by ROS, which oxidize amino acid side chains and lead to the formation of protein carbonyls, impairing protein structure and function. These oxidative modifications can affect enzymes, structural proteins, and signaling molecules, disrupting cellular homeostasis. In the vasculature, oxidative stress-induced protein damage contributes to endothelial dysfunction by impairing the activity of enzymes that regulate vascular tone and integrity. For example, oxidatively modified eNOS produces less NO and more superoxide, further exacerbating vascular dysfunction [40]. ROS can also cause DNA damage, including single- and double-strand breaks, as well as oxidative modifications to nucleotide bases. One of the most common oxidative DNA lesions is 8-hydroxy-2'-deoxyguanosine (8-OHdG), which is often used as a biomarker of oxidative DNA damage. Persistent DNA damage activates DNA damage response (DDR) pathways, leading to cellular senescence or apoptosis. Senescent cells, particularly in the vasculature, adopt a senescence-associated secretory phenotype (SASP), characterized by the release of pro-inflammatory cytokines, chemokines, growth factors, and matrix metalloproteinases (MMPs). These factors further promote inflammation, disrupt the extracellular matrix (ECM), and contribute to vascular remodeling, accelerating vascular aging [41].

In the following sections, we will explore the role of mitochondria, autophagy, and the mechanisms involving sirtuin-1 (SIRT1), NOX, and eNOS in the disruption of redox homeostasis during senescence

### 3.1.1 Mitochondria and Impaired Autophagy

Mitochondrial dysfunction is a central pathogenic driver in vascular inflammaging, significantly contributing to age-related vascular diseases [42]. Mitochondria are essential for cellular energy production and homeostasis, but their DNA is particularly vulnerable to oxidative stress due to its proximity to the electron transport chain and the high levels of ROS generated during oxidative phosphorylation. Consequently, mitochondrial DNA (mtDNA) mutations and deletions can arise, impairing mitochondrial function, reducing ATP production, and leading to an excess of ROS [43]. These dysfunctional mitochondria release damaged mtDNA into the cytoplasm and extracellular space, where it acts as damage-associated molecular patterns (DAMPs). These molecules are recognized by pattern recognition receptors (PRRs), such as toll-like receptors (TLRs) and nucleotide-binding oligomerization domain-like receptors (NLRs), triggering inflammasome activation. This leads to the cleavage of pro-interleukin-1β (pro-IL-1β) and pro-interleukin-18 (pro-IL-18) into their active forms, which are then secreted to amplify the inflammatory response [44]. This chronic inflammatory state, characterized by sustained immune activation, results in endothelial dysfunction, increased vascular stiffness, and the development of atherosclerosis—hallmarks of vascular aging [45].

Mitophagy, the selective autophagy and degradation of damaged mitochondria, is a critical mechanism for mitigating mtDNA damage-induced inflammaging [42]. This process is primarily governed by the PINK1-parkin pathway, where PINK1 accumulates on the outer membrane of damaged mitochondria, recruiting the E3 ubiquitin ligase parkin, tagging the mitochondria for degradation by lysosomes [46]. Efficient mitophagy ensures the removal of dysfunctional mitochondria, reducing ROS levels and preventing the release of pro-inflammatory mitochondrial components into the cytoplasm, which could activate inflammasomes and NF-κB signaling pathways, leading to vascular inflammation [47]. However, during aging, the efficiency of mitophagy declines due to downregulation of autophagy-related genes and proteins, as well as diminished cellular responsiveness to mitophagic signals. This age-related decline in mitophagy contributes to the accumulation of damaged mitochondria, exacerbating oxidative stress and inflammaging [48]. Thus, therapeutic strategies aimed at enhancing mitophagy or modulating its upstream regulators—such as sirtuins, which influence cellular metabolism and stress responses—hold potential in addressing the mitochondrial contributions to vascular aging and related inflammatory diseases [49].

### 3.1.2 SIRT1 Functionality

SIRT1, a NAD+-dependent deacetylase, plays a pivotal role in regulating inflammaging within the vasculature. SIRT1 exerts its anti-inflammatory effects primarily through the deacetylation of transcription factors and co-regulators [50]. By deacetylating NF-κB, SIRT1 decreases the expression of pro-inflammatory cytokines and adhesion molecules, thus reducing endothelial inflammation and preserving vascular integrity. Additionally, SIRT1 activation promotes the expression of antioxidant enzymes and nitric oxide synthase (NOS), lowering oxidative stress and enhancing vasodilation [51]. SIRT1 also influences cellular senescence, a key component of aging, by deacetylating and activating substrates involved in DNA repair and stress resistance, such as the forkhead box O (FOXO) transcription factors. These factors are critical for promoting cellular longevity and protecting against apoptosis [52]. Furthermore, SIRT1 stabilizes telomeres, delaying cellular senescence and enhancing the survival and function of endothelial cells [53].

A crucial part of the protective mechanism of SIRT1 is its interaction with nuclear factor erythroid 2-related factor 2 (Nrf2), a transcription factor that regulates the expression of antioxidant proteins essential for protecting against oxidative damage caused by injury and inflammation [54]. SIRT1 activates Nrf2 via direct deacetylation, enabling Nrf2 to translocate into the nucleus and bind to antioxidant response elements (AREs) in DNA. This activation leads to the upregulation of genes responsible for detoxification and antioxidant defense, thereby mitigating oxidative stress and reducing the inflammatory response in the vasculature [55].

### 3.1.3 NOX Activity

NOX play a crucial role in driving vascular inflammaging by producing ROS, which function both as signaling molecules and agents of oxidative damage [56]. The NOX enzyme family, particularly the NOX2 and NOX4 isoforms, are upregulated in aged vascular tissues, activated by upstream stimuli such as angiotensin II, pro-inflammatory cytokines, and advanced glycation end-products (AGEs) [57]. These stimuli trigger the assembly of NOX subunits at the cell membrane, facilitating the transfer of electrons from NADPH to molecular oxygen, producing O_2_^•-^ [58]. These O_2_^•-^ quickly dismutate to H_2_O_2_, further contributing to oxidative stress. NOX-derived ROS causes endothelial dysfunction through several downstream mechanisms. They reduce NO bioavailability by promoting NO degradation and facilitating the formation of peroxynitrite (ONOO^-^), a potent oxidant. This results in impaired vasodilation and increased vascular stiffness [59]. Additionally, ROS activate redox-sensitive transcription factors such as NF-κB, which enhance the expression of pro-inflammatory cytokines and adhesion molecules, promoting leukocyte recruitment and adhesion to the endothelium. This perpetuates local inflammation and accelerates atherosclerotic plaque formation. Excessive ROS levels also stimulate the release of MMPs, leading to the degradation of the ECM and vascular remodeling [60]. Moreover, NOX-derived ROS induce oxidative modifications of lipids, proteins, and DNA, exacerbating cellular senescence and fostering the SASP in vascular cells. The SASP, characterized by the secretion of pro-inflammatory cytokines, chemokines, and proteases, amplifies inflammation and contributes to structural remodeling [61]. As a result, therapeutic strategies targeting NOX activity—such as NOX inhibitors, antioxidants, and angiotensin II receptor blockers (ARBs)—offer the potential to reduce NOX-induced oxidative stress and inflammaging in the vasculature, improving vascular health and reducing the risk of cardiovascular diseases in the aging population [62].

**Figure 1.**
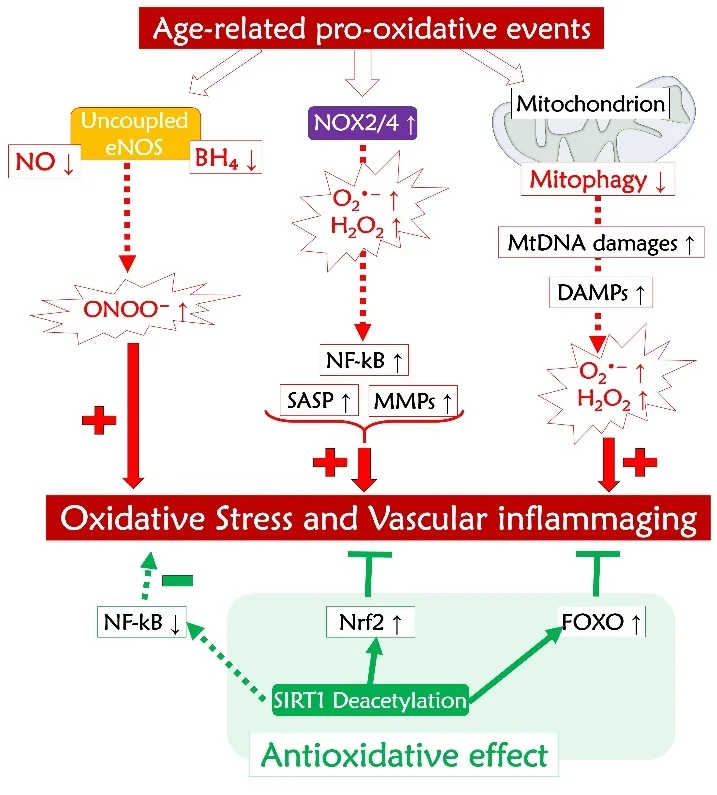
**Overview on the main molecular pathways involving oxidative stress and contributing to the process of vascular inflammaging**. Abbreviations: BH_4_, tetrahydrobiopterin; DAMPs, damage-associated molecular patterns; eNOS, endothelial nitric oxide synthase; FOXO, forkhead box O; H_2_O_2_, hydrogen peroxide; MMPs, matrix metalloproteinases; MtDNA, mitochondrial DNA; NF-κB, nuclear factor kappa-light-chain-enhancer of activated B cells; NO, nitric oxide; NOX2/4, NADPH oxidase2/4; Nrf2, nuclear factor erythroid 2-related factor 2; O_2_^•-^, superoxide anions; ONOO^-^, peroxynitrite; SASP, senescence-associated secretory phenotype; SIRT1, sirtuin-1.

### 3.1.4 The eNOS Pathway

eNOS plays a pivotal role in maintaining vascular health, particularly in the context of inflammaging [63]. The activity of eNOS can be enhanced through phosphorylation via the phosphoinositide 3-kinase (PI3K)/Akt pathway [64]. eNOS generates NO, a potent vasodilator and anti-inflammatory molecule, and its function is highly dependent on the availability of the cofactor tetrahydrobiopterin (BH4) [37]. In healthy conditions, eNOS utilizes BH4 to convert L-arginine into NO and L-citrulline, ensuring efficient NO production, which is critical for maintaining endothelial function and vascular tone [65]. However, when BH4 levels are insufficient, eNOS becomes uncoupled, producing O_2_^•-^ instead of NO. This shift increases oxidative stress and contributes to endothelial dysfunction and vascular inflammation. Oxidative stress and inflammatory cytokines further exacerbate this process by depleting BH4 and converting it into dihydrobiopterin (BH2), which is less effective at supporting eNOS activity [66]. This creates a vicious cycle, where increased ROS production depletes BH4, leading to further eNOS uncoupling, reduced NO bioavailability, and elevated O_2_^•-^ production. This feedback loop propagates vascular inflammation and oxidative damage, worsening the effects of inflammaging [67] (Fig. 1).

### 3.2 Inflammatory Status and Immunosenescence

Immunosenescence, along with the senescence of immune cells, plays a pivotal role in the development of vascular inflammaging, driven by distinct molecular mechanisms and cytokine signaling pathways [68]. Immunosenescence involves the gradual decline of the adaptive immune system, especially T cell function. Persistent antigenic exposure, particularly from chronic infections like cytomegalovirus (CMV), leads to T cell exhaustion and the accumulation of senescent T cells [69]. These senescent T cells, particularly CD28null T cells, show diminished proliferative capacity and an altered cytokine profile characterized by increased secretion of pro-inflammatory cytokines such as TNF-α, IL-6, and interferon-gamma (IFN-γ). This pro-inflammatory environment contributes to systemic low-grade inflammation, a key feature of inflammaging [70]. The upstream drivers of this process include activation of major inflammatory pathways such as NF-κB and p38 mitogen-activated protein kinase (MAPK) in response to oxidative stress and chronic antigenic stimulation [71]. The NF-κB signaling pathway is particularly significant in mediating inflammaging within the vasculature, as it regulates immune and inflammatory responses that accelerate vascular aging and the progression of vascular diseases [72]. NF-κB activation is triggered by various stimuli, including oxidative stress, cytokines, and pathogens [73]. Once activated, NF-κB translocates to the nucleus, where it promotes the transcription of numerous pro-inflammatory genes, including cytokines, chemokines, adhesion molecules, and enzymes such as cyclooxygenase-2 (COX-2) and inducible nitric oxide synthase (iNOS) [74]. These gene products exacerbate vascular inflammation, contributing to endothelial dysfunction, increased vascular permeability, and leukocyte adhesion to the endothelium, perpetuating the cycle of inflammaging [75]. Importantly, NF-κB activation is tightly regulated by inhibitors such as IκB proteins, which sequester NF-κB in the cytoplasm under non-stimulated conditions [76]. Therapies targeting NF-κB activation, including upstream kinase inhibitors, modulators of IκB degradation, and antioxidants that reduce oxidative stress, are being explored to mitigate chronic vascular inflammation and promote vascular health in aging populations [73].

Closely linked to NF-κB activation is the NOD-like receptor pyrin domain-containing protein 3 (NLRP3) inflammasome, a key mediator of pyroptosis in vascular tissues. The NLRP3 inflammasome orchestrates responses to cellular stress, metabolic disturbances, and DAMPs [77]. Upon activation, NLRP3 recruits apoptosis-associated speck-like proteins containing a caspase recruitment domain (ASC) and procaspase-1, leading to the cleavage of procaspase-1 into its active form, caspase-1 [78]. This cascade is critical for the maturation and secretion of pro-inflammatory cytokines IL-1β and IL-18, which are major contributors to the inflammatory environment of vascular aging [79]. Mitochondrial dysfunction, characterized by the release of mtDNA and ROS, is a potent activator of the NLRP3 inflammasome. Additionally, extracellular ATP and cholesterol crystals, particularly in atherogenic conditions, can stimulate NLRP3 activation [80]. The downstream effects of NLRP3 activation include endothelial dysfunction, upregulation of adhesion molecules, and immune cell infiltration into the vascular wall, exacerbating inflammation and promoting the progression of atherosclerosis and other age-related vascular diseases [81]. A hallmark of inflammaging is the switch to the SASP. SASP is a key marker of vascular inflammaging, characterized by the secretion of pro-inflammatory cytokines, chemokines, growth factors, and proteases by senescent cells [82]. This secretory profile not only influences the local cellular environment but also has systemic effects that contribute to age-related vascular diseases such as atherosclerosis, hypertension, and endothelial dysfunction [83]. The initiation of SASP is regulated by DDR pathways, which activate p53, p21, and p16, leading to cell cycle arrest and the subsequent development of SASP [84]. Key mediators such as NF-κB and inflammasomes play critical roles in amplifying the inflammatory response through SASP. NF-κB, for instance, acts as a transcription factor for many SASP components, enhancing their expression in response to oxidative stress and other aging-related stimuli [84]. The downstream effects of SASP include immune cell recruitment to the endothelium, disruption of endothelial integrity, and increased vascular permeability, all of which intensify vascular aging and related pathologies [83]. SASP factors such as IL-6 and TNF-α can also induce paracrine senescence, spreading the aging phenotype to neighboring cells and amplifying the inflammatory response within vascular tissues [85]. Targeting SASP or its key components offers significant therapeutic potential for combating vascular aging. Promising strategies include the use of senolytics to eliminate senescent cells, inhibitors to block key SASP factors or pathways, and antioxidants to reduce oxidative stress, thereby inhibiting SASP-inducing mechanisms [86].

### 3.3 Fibrotic Events during Aging

The transforming growth factor-beta (TGF-β)/suppressor of mothers against decapentaplegic (SMAD) signaling pathway plays a crucial role in regulating vascular aging and inflammaging, influencing cellular responses through both canonical and non-canonical mechanisms. This pathway is central to the regulation of cellular proliferation, differentiation, apoptosis, and fibrosis, processes that become increasingly dysregulated with age [87]. TGF-β, a multifunctional cytokine produced by various cell types, including endothelial cells and macrophages, binds to its serine/threonine kinase receptors (TGF-βRI and TGF-βRII), initiating phosphorylation of receptor-regulated SMADs (R-SMADs). These R-SMADs form complexes with SMAD4, translocating to the nucleus to regulate gene expression linked to ECM production, angiogenesis, and inflammatory modulation [88]. In vascular inflammaging, TGF-β/SMAD signaling contributes to pathological remodeling by promoting ECM synthesis and deposition, increasing tissue stiffness, and advancing conditions such as atherosclerosis and hypertension [89]. The pathway also induces endothelial-to-mesenchymal transition (EndMT) under stress, worsening vascular dysfunction and enhancing fibrosis and vascular permeability [90]. Furthermore, TGF-β modulates immune responses by regulating the differentiation and activity of immune cells, including T cells and macrophages, thereby influencing inflammation within the vascular wall [91].

In fibrotic events, MMPs, a family of zinc-dependent endopeptidases, play a pivotal role in vascular inflammaging by mediating ECM remodeling [92]. The activity of MMPs in aging vessels is regulated by multiple upstream signals, including oxidative stress, cytokines like TNF-α and IL-1, and mechanical stress, all of which are prevalent in the aging vasculature [93]. MMPs, particularly MMP-2 and MMP-9, are upregulated in response to these stimuli, leading to the degradation of collagen and elastin, which results in thinning and stiffening of vascular walls, a hallmark of vascular aging [94]. Additionally, MMPs contribute to the migration and activation of inflammatory cells in the vascular wall by cleaving matrix components and non-matrix substrates, such as chemokines, cytokines, and cell surface receptors, which amplify the inflammatory response and accelerate vascular damage [95]. Normally, the activity of MMPs is counterbalanced by tissue inhibitors of metalloproteinases (TIMPs); however, in aging vessels, the balance shifts towards a pro-degradative state, leading to increased ECM degradation. This imbalance contributes to pathological outcomes such as aneurysm formation, plaque instability in atherosclerosis, and increased vascular permeability [96].

### 3.4 Impact of the Renin-Angiotensin-Aldosterone System

The renin-angiotensin-aldosterone system (RAAS) plays a crucial role in the pathophysiology of vascular inflammaging, contributing significantly to the changes observed in vascular aging [97]. Angiotensin II, one of the key active components of the RAAS, is a potent vasoconstrictor that also stimulates the angiotensin II type 1 receptor (AT1R), triggering the production of pro-inflammatory cytokines [98]. These inflammatory mediators are implicated in endothelial dysfunction, a hallmark of vascular aging. Additionally, angiotensin II promotes the expression of adhesion molecules, facilitating leukocyte adhesion and infiltration into the vascular wall, thereby enhancing the inflammatory response [99]. RAAS activation also leads to the upregulation of NOX, increasing oxidative stress, which in turn reduces NO bioavailability and stimulates the production of ROS [100, 101]. This cascade of events exacerbates endothelial damage and accelerates cellular senescence, contributing to increased vascular stiffness and the formation of atherosclerotic plaques [102]. Furthermore, aldosterone, another component of the RAAS, contributes to vascular inflammation and remodeling by activating mineralocorticoid receptors in vascular smooth muscle and endothelial cells, further promoting inflammation [98].

### 3.5 The AGE/RAGE Axis

**Figure 2.**
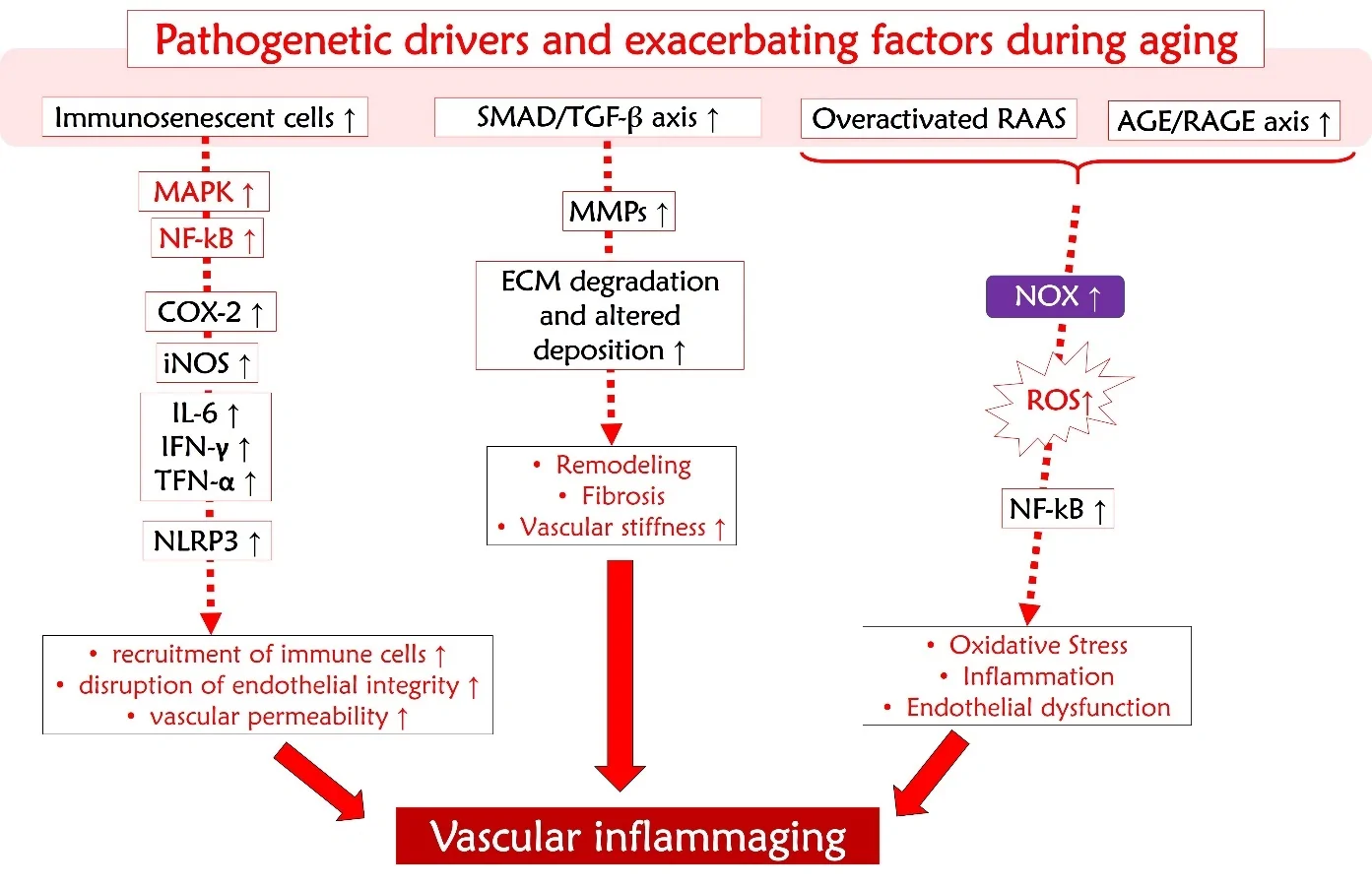
**Schema on the central pathogenetic drivers of inflammation and exacerbating factors involved in vascular inflammaging**. Abbreviations: AGEs, advanced glycation end-products; COX-2, cyclooxygenase-2; ECM, extracellular matrix; IFN-γ, interferon-gamma; IL-6, interleukin-6; iNOS, inducible nitric oxide synthase; MAPK, mitogen-activated protein kinase; MMPs, matrix metalloproteinases; NF-κB, nuclear factor kappa-light-chain-enhancer of activated B cells; NLRP3, NOD-like receptor pyrin domain-containing protein 3; NOX, NADPH oxidases; RAAS, renin-angiotensin-aldosterone system; RAGE, receptor for advanced glycation end products; ROS, reactive oxygen species; SMAD, suppressor of mothers against decapentaplegic; TGF-β, transforming growth factor-beta; TNF-α, tumor necrosis factor-alpha.

The Advanced Glycation End Products (AGE)/Receptor for Advanced Glycation End Products (RAGE) axis plays a critical role in the metabolic disturbances associated with hyperglycemia, diabetes, and hyperlipidemia [103]. AGEs, formed through non-enzymatic reactions between sugars and proteins or lipids, accumulate more rapidly under hyperglycemic conditions typical of diabetes. These AGEs bind to RAGE, a cell surface receptor belonging to the immunoglobulin superfamily, triggering signaling cascades that enhance inflammatory responses [104]. The AGE-RAGE interaction activates key transcription factors, such as NF-κB, which drive the expression of pro-inflammatory cytokines, adhesion molecules, and other mediators that contribute to endothelial dysfunction. This dysfunction manifests as increased oxidative stress, reduced NO bioavailability, and enhanced vascular smooth muscle cell proliferation and migration, all of which are key aspects of vascular aging [105]. Hyperlipidemia further exacerbates this process by raising levels of oxidized low-density lipoprotein (ox-LDL), which also interacts with RAGE, intensifying inflammatory signaling. The combined effects of AGEs and ox-LDL engaging RAGE create a chronic inflammatory environment, characterized by endothelial dysfunction, increased vascular stiffness, and atherogenesis, thereby accelerating vascular inflammaging [106]. The persistent activation of these inflammatory pathways, driven by the AGE/RAGE interaction, highlights its pivotal role in vascular aging and underscores the potential therapeutic value of targeting the AGE/RAGE axis to reduce the burden of cardiovascular diseases linked to inflammaging [107] (Fig. 2).

## 4. Vascular Inflammaging in Individual Age-Related Diseases

### 4.1 Inflammaging in Cardiovascular Diseases

#### 4.1.1 Inflammaging in Arterial Hypertension

Mechanisms driving vascular dysfunction and increased systemic vascular resistance, influenced by inflammaging, have a significant impact on hypertension. Current research shows that the chronic low-grade inflammation associated with aging contributes to endothelial dysfunction, a critical factor in the development of hypertension [35]. This dysfunction is marked by reduced bioavailability of vasodilators like NO and an increase in vasoconstrictors such as endothelin-1 [108]. Inflammaging also induces vascular remodeling, where arterial walls thicken and stiffen due to increased deposition of collagen and other matrix proteins [109]. These changes lead to decreased arterial compliance and elevated pulse wave velocity, which are strongly linked to the higher systolic blood pressure commonly observed in elderly hypertensive patients [110]. In addition, inflammaging promotes conditions that encourage sodium retention, worsening fluid volume and pressure increases that further aggravate hypertension [111]. Elevated levels of inflammatory markers such as CRP, IL-6, and TNF-α have been found in hypertensive patients, correlating with both disease severity and progression [112]. These cytokines drive vascular inflammation by activating NF-κB, a transcription factor that regulates the expression of numerous pro-inflammatory genes. NF-κB activation in vascular endothelial cells stimulates the production of adhesion molecules and chemokines, attracting monocytes to the endothelium and promoting their transformation into macrophages, creating a cycle of inflammation and endothelial damage [113]. Furthermore, the angiotensin II pathway, essential in blood pressure regulation, also intensifies vascular inflammation by promoting ROS production and reducing NO availability, which diminishes endothelium-dependent vasodilation [114].

#### 4.1.2 Inflammaging in Myocardial Infarction

Inflammaging plays a crucial role in the pathophysiology of myocardial infarction (MI) by intensifying the underlying mechanisms that lead to cardiac injury. In elderly individuals, this process is characterized by chronic systemic inflammation, which drives structural and functional changes within the cardiovascular system [2]. Key pathophysiological alterations include increased arterial stiffness, endothelial dysfunction, and a heightened atherosclerotic burden, all of which create conditions conducive to thrombosis and plaque rupture, thereby predisposing the myocardium to ischemic events [115]. Following a MI, the pro-inflammatory environment associated with inflammaging impairs the cardiac healing process, promoting maladaptive remodeling and the development of heart failure. Chronic inflammation interferes with normal repair mechanisms, resulting in excessive fibrotic scarring and diminished myocardial function, which together worsen outcomes in elderly patients [116]. At the molecular level, elevated levels of IL-6 and TNF-α have been linked to the progression of atherosclerosis and plaque instability, key precursors to MI [117]. Additionally, studies have demonstrated that SASP factors, such as IL-1β, IL-18, and TGF-β, further contribute to adverse cardiac remodeling by promoting fibroblast proliferation and collagen deposition in the myocardium [118]. These cytokines also activate inflammatory signaling pathways, such as NF-κB and signal transducer and activator of transcription 3 (STAT3), which not only perpetuate chronic inflammation but also hinder the reparative capacity of cardiac tissue post-infarction [119].

#### 4.1.3 Inflammaging in Aortic Aneurysm Formation

**Figure 3.**
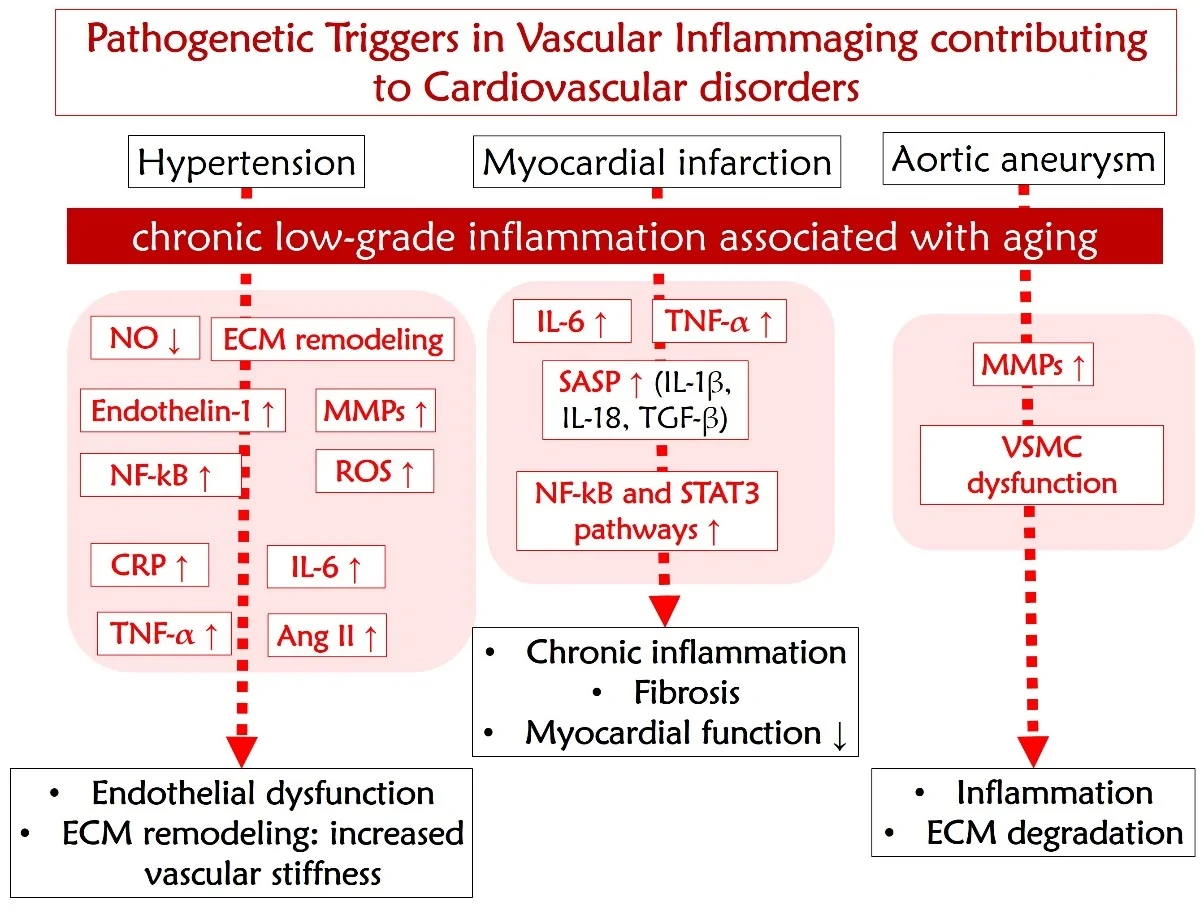
**Illustration of the pivotal pathogenetic pathways contributing to hypertension, myocardial infarction and aortic aneurism, in terms of mechanisms of vascular inflammaging for cardiovascular disorders**. Abbreviations: Ang II, angiotensin II; CRP, C-reactive protein; ECM, extracellular matrix; IL-6, interleukin-6; IL-1β, interleukin-1β; IL-18, interleukin-18; MMPs, matrix metalloproteinases; NF-κB, nuclear factor kappa-light-chain-enhancer of activated B cells; NO, nitric oxide; ROS, reactive oxygen species; SASP, senescence-associated secretory phenotype; STAT3, signal transducer and activator of transcription 3; TGF-β, transforming growth factor-beta; TNF-α, tumor necrosis factor-alpha; VSMC, vascular smooth muscle cell.

The development of aortic aneurysms, characterized by abnormal dilation of the aorta, is significantly influenced by inflammaging. This condition is largely driven by degenerative changes in the aortic wall, mediated by chronic low-grade inflammation [120]. A key mechanism through which inflammaging contributes to aortic aneurysm formation is the disruption of vascular smooth muscle cells (VSMCs) and ECM degradation. Chronic inflammatory stimuli cause VSMCs to lose their contractile phenotype and adopt a synthetic phenotype [121]. This phenotypic shift is crucial as it leads to increased production of MMPs, particularly MMP-2 and MMP-9, which degrade elastin and collagen, critical structural components of the aortic wall, thereby weakening the vessel and predisposing it to dilation and aneurysm formation [122]. In addition to MMP-mediated ECM degradation, the recruitment and infiltration of inflammatory cells, such as macrophages and lymphocytes, into the aortic wall further aggravates the inflammatory response. These immune cells release cytokines that stimulate VSMCs, promoting further secretion of MMPs and amplifying ECM breakdown [123]. This self-perpetuating cycle of inflammation and ECM degradation is a hallmark of the pathophysiological changes observed in aortic aneurysms [124] (Fig. 3).

### 4.2 Inflammaging in Cerebrovascular Diseases

#### 4.2.1 Inflammaging in Stroke

Inflammaging profoundly affects the cerebrovascular system, particularly influencing stroke pathophysiology through age-related mechanisms that exacerbate risk and outcome [125]. The chronic systemic inflammation characteristic of inflammaging drives both structural and functional changes in cerebral arteries [126, 127]. These alterations include arterial remodeling, marked by increased wall thickness and decreased elasticity, which not only predispose individuals to ischemic events but also impair cerebral autoregulation [128]. Impaired autoregulation compromises the brain's ability to adjust blood flow during neuronal activity or in response to systemic blood pressure fluctuations, heightening the risk of both ischemic and hemorrhagic strokes [129]. Moreover, inflammaging is linked to BBB dysfunction, where increased permeability due to inflammatory mediators allows leukocytes and neurotoxic substances to penetrate the brain parenchyma, intensifying neuronal damage during and after stroke [130]. Mechanistically, stroke-related inflammaging is characterized by elevated systemic levels of pro-inflammatory cytokines, along with increased activation of peripheral immune cells that infiltrate the brain. These cytokines and immune cells amplify local inflammation, exacerbating neuronal injury [131]. The role of microglial cells, the brain’s resident immune cells, is critical in this process. In response to aging and systemic inflammatory signals, microglia become activated and often adopt a pro-inflammatory phenotype [132]. This activation not only directly contributes to neuronal damage by releasing inflammatory mediators and excitotoxic agents but also impairs the brain’s ability to repair and regenerate after ischemic injury [133]. Furthermore, chronic activation of the complement system, a key component of the innate immune response, becomes dysregulated with age, leading to synaptic loss and neuronal death, thereby contributing to post-stroke cognitive decline [134].

#### 4.2.2 Inflammaging in Vascular Dementia

Vascular dementia, a common neurocognitive disorder arising from cerebrovascular damage and brain ischemia, is profoundly influenced by inflammaging [135]. In older adults, chronic low-grade inflammation accelerates the progression of atherosclerosis and small vessel disease, which severely compromises cerebral perfusion and leads to prolonged hypoxia [136]. This reduced blood flow fosters the development of cerebral microinfarcts and extensive white matter lesions, directly impairing cognitive function [137]. Inflammaging also contributes to the breakdown of the BBB, permitting peripheral inflammatory mediators to enter the brain, further promoting neuroinflammation and neuronal injury [138]. These pathological changes are exacerbated by cerebral amyloid angiopathy, which is worsened by inflammaging and adds to the complex pathology of vascular dementia by disrupting cerebral blood flow and heightening susceptibility to hemorrhagic strokes [139]. At the molecular level, vascular dementia associated with inflammaging is characterized by elevated levels of pro-inflammatory cytokines, sustaining chronic inflammation in both cerebral vasculature and neural tissue [140]. These cytokines drive endothelial dysfunction, increase BBB permeability, and activate glial cells, which in turn release additional inflammatory mediators and generate ROS [135, 141]. This inflammatory cascade is regulated by key signaling pathways, including NF-κB and MAPKs, which are crucial in the transcriptional activation of inflammatory genes [142]. Chronic activation of these pathways disrupts neuronal and synaptic function, contributing significantly to the cognitive decline characteristic of vascular dementia. Additionally, the accumulation of AGEs and the RAGE in aging vasculature perpetuates inflammatory processes, further linking inflammaging to the pathogenesis of vascular dementia [143].

#### 4.2.3 Inflammaging in Alzheimer's Disease

**Figure 4.**
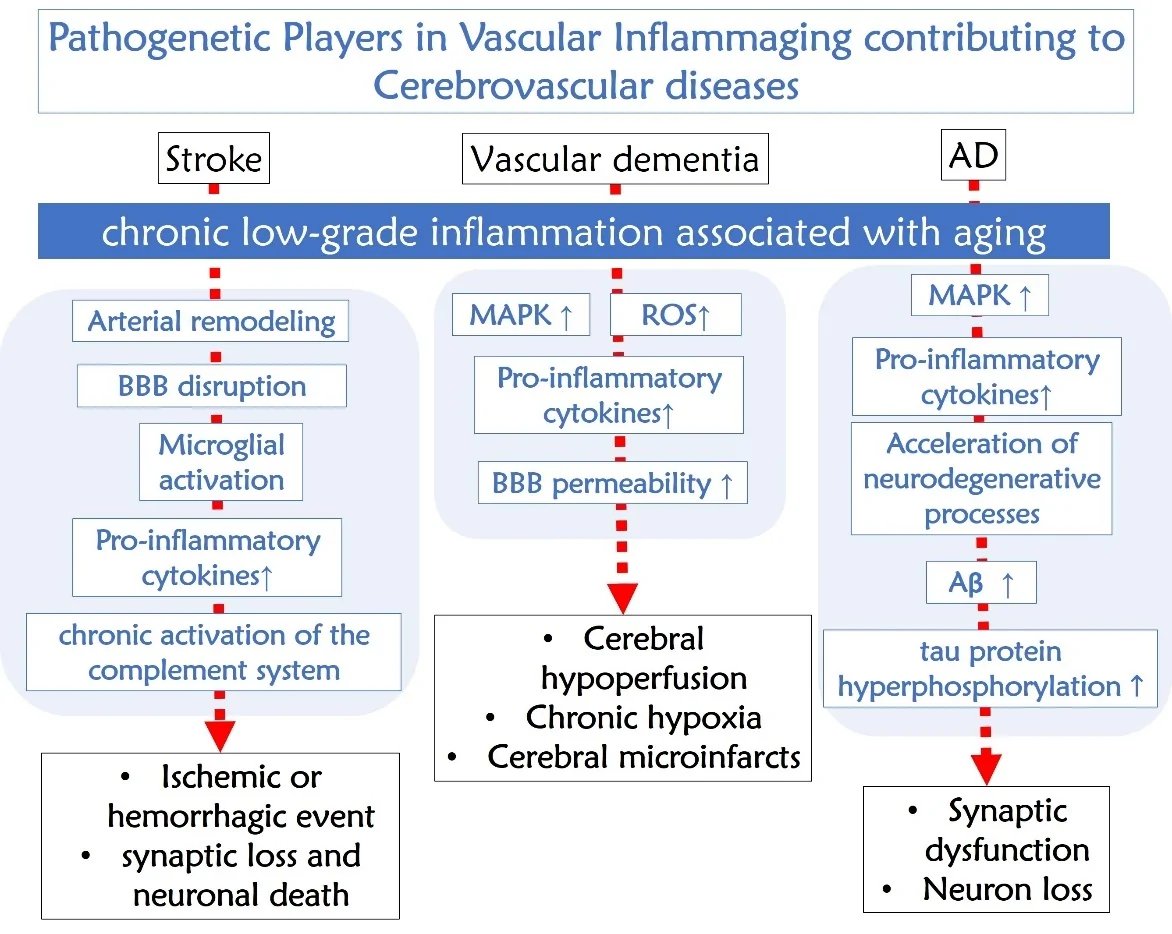
**Schematic overview on the main molecular pathomechanisms associated with vascular inflammaging, in cerebrovascular diseases such as stroke, vascular dementia and Alzheimer’s disease**. Abbreviations: Aβ, amyloid-beta; AD, Alzheimer's disease; BBB, blood-brain barrier; MAPK, mitogen-activated protein kinase; ROS, reactive oxygen species.

Alzheimer's disease (AD), a neurodegenerative disorder characterized by cognitive decline and memory impairment, is closely linked to chronic low-grade inflammation, a hallmark of both aging and the disease itself. Inflammaging plays a pivotal role in disrupting cellular homeostasis and accelerating neurodegenerative processes by impacting neuronal cells and altering the brain’s microenvironment [144]. This chronic inflammation exacerbates the accumulation of amyloid-beta (Aβ) plaques and tau protein hyperphosphorylation, which impair neuronal communication—key pathological features of AD [145]. Additionally, inflammaging promotes the sustained activation of microglia, the brain's resident immune cells, resulting in persistent neuroinflammation. This chronic inflammatory state contributes to synaptic dysfunction and progressive neuronal loss. The breakdown of the BBB, a consequence of aging-related inflammation, further allows peripheral immune cells to infiltrate the brain, worsening local inflammation and advancing the pathology of AD [146]. At the molecular level, inflammaging in AD is driven by a complex network of pro-inflammatory cytokines and signaling pathways that enhance inflammatory responses within the central nervous system [147]. These cytokines activate key signaling cascades, notably NF-κB and MAPKs, which perpetuate the expression of inflammatory genes, intensify cellular stress, and promote apoptosis [148] (Fig. 4).

### 4.3 Inflammaging in Ocular Vascular Pathologies

Inflammaging in ocular blood vessels drives complex pathophysiological changes that progressively impair ocular function, particularly affecting the retina and uvea, which are crucial for visual acuity. A key feature of these changes is the thickening of the basement membrane, which increases vascular rigidity and decreases elasticity, directly hindering the exchange of nutrients and metabolic waste between the blood vessels and retinal tissue [149]. This disruption leads to the accumulation of toxic substances that may damage retinal cells [12]. Another critical change is the loss of pericytes, cells that are vital for maintaining capillary stability and regulating endothelial cell proliferation. The depletion of pericytes contributes to vascular instability and raises the risk of microaneurysms, commonly seen in DR [150]. The decline in pericyte function is often driven by an environment rich in pro-inflammatory cytokines and oxidative stress, which further exacerbates vascular damage [151].

Endothelial dysfunction is a central element of inflammaging in the eye. As endothelial cells lining the inner walls of blood vessels become progressively dysfunctional with age, their ability to produce NO diminishes. This deficiency contributes to endothelial activation, fostering a pro-thrombotic and pro-inflammatory state within the ocular vasculature [152]. The situation is aggravated by the increased expression of adhesion molecules, which promote leukocyte adhesion to endothelial cells, enabling inflammatory cells to infiltrate surrounding retinal tissues [153]. Increased vascular permeability also plays a pivotal role in the pathology of inflammaging-related ocular diseases. The heightened permeability allows plasma components to leak into retinal tissues, contributing to macular edema, a significant complication in conditions like AMD and DR [13, 154]. Additionally, the breakdown of the blood-retinal barrier (BRB), which normally protects the retina from circulating toxins and pathogens, occurs due to both increased vascular permeability and the disintegration of tight junctions between endothelial cells [155]. Collectively, these pathophysiological changes create a chronic, low-grade inflammatory environment within the eye, accelerating cellular aging and leading to the progressive decline in retinal function [156].

#### 4.3.1 Inflammaging in Age-Related Macular Degeneration

AMD is a leading cause of vision impairment in the elderly, and inflammaging plays a central role in its pathogenesis. In AMD, inflammaging contributes to macular degeneration through several interconnected mechanisms. The early pathological hallmark is the accumulation of drusen, extracellular deposits rich in lipids and proteins, between the retinal pigment epithelium (RPE) and Bruch’s membrane [157]. These deposits trigger an immune response, activating local microglia and macrophages, which release pro-inflammatory cytokines such as IL-6, TNF-α, and complement proteins C3 and C5a [158]. Additionally, oxidative stress and mitochondrial dysfunction in retinal cells contribute to the inflammatory environment by producing ROS, which further damage the RPE and photoreceptors, accelerating degeneration [153]. Inflammation-induced upregulation of vascular endothelial growth factor (VEGF) promotes choroidal neovascularization, a major cause of vision loss in the neovascular form of AMD. The chronic inflammatory state is also characterized by the activation of the complement system, with genetic variations in complement factor H (CFH) influencing individual susceptibility to AMD [159]. Moreover, the NLRP3 inflammasome becomes upregulated, enhancing the production of IL-1β and IL-18, potent inflammatory mediators that further contribute to retinal cell damage [160]. The ongoing inflammation and tissue damage also disrupt the BRB, allowing the infiltration of additional inflammatory cells, which exacerbates the damage.

These molecular and cellular mechanisms highlight the critical role of inflammaging in AMD, driving both early and late stages of the disease through sustained inflammation that damages retinal structures and impairs visual function [161].

#### 4.3.2 Inflammaging in Diabetic Retinopathy

**Figure 5.**
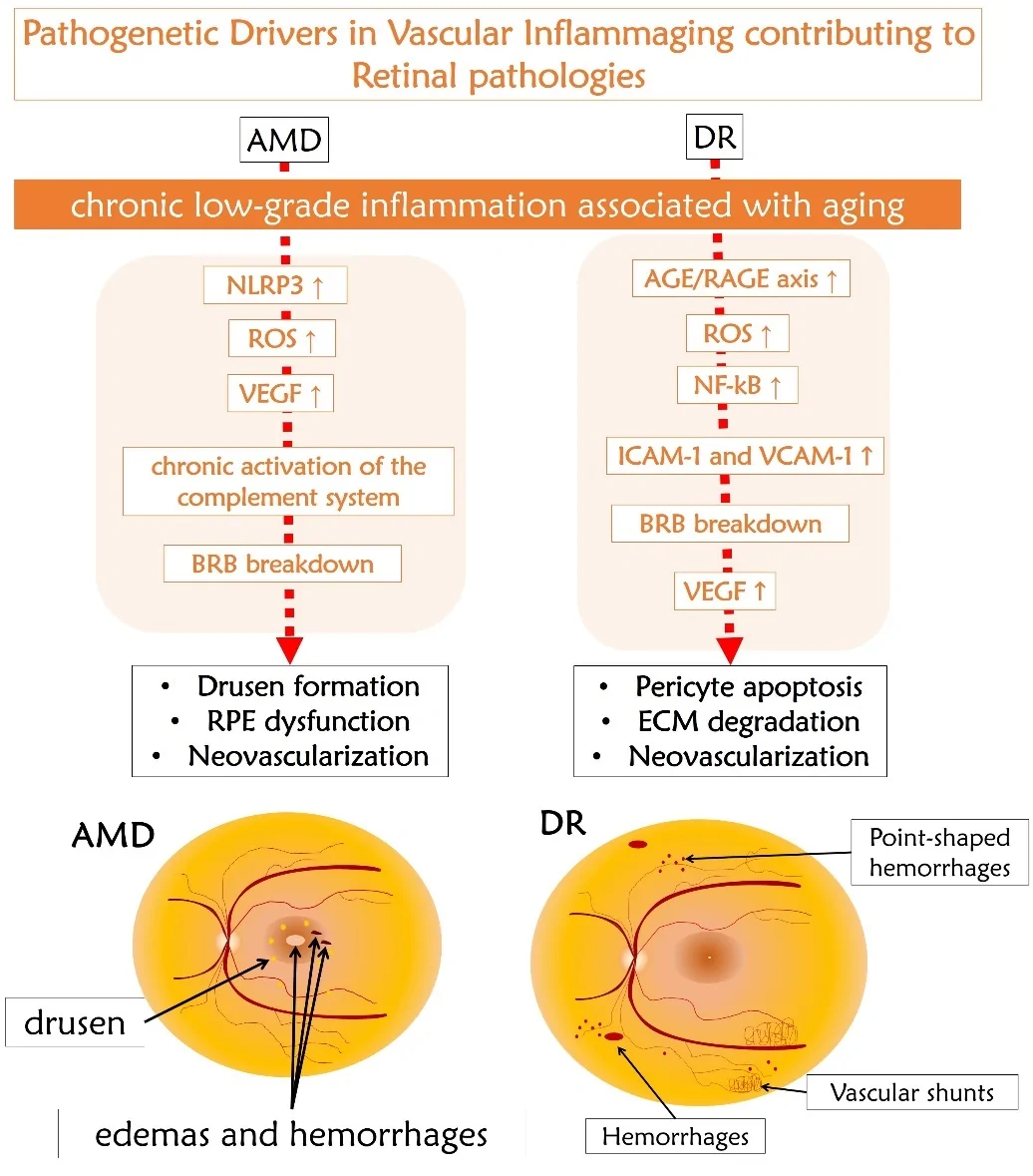
**Representation of the central pathogenetic players linked with vascular inflammaging, in highly prevalent and invalidating retinal disorders like AMD and DR**. Abbreviations: AGEs, advanced glycation end-products; AMD, age-related macular degeneration; BRB, blood-retinal barrier; DR, diabetic retinopathy; ECM, extracellular matrix; ICAM-1, intercellular adhesion molecule-1; NF-κB, nuclear factor kappa-light-chain-enhancer of activated B cells; NLRP3, NOD-like receptor pyrin domain-containing protein 3; RAGE, receptor for advanced glycation end products; ROS, reactive oxygen species; RPE, retinal pigment epithelium; VCAM-1, vascular cell adhesion molecule-1; VEGF, vascular endothelial growth factor.

Inflammaging plays a key role in the progression of DR by exacerbating vascular dysfunction and advancing microvascular complications. The process is triggered by hyperglycemia-induced metabolic disturbances, leading to an increase in AGEs and ROS [162]. These pro-inflammatory stimuli activate NF-κB signaling pathways, which result in the upregulation of cytokines like IL-6, TNF-α, and IL-1β. This persistent activation contributes to endothelial dysfunction, marked by decreased NO bioavailability and heightened vascular permeability [163]. Additionally, inflammaging enhances the expression of vascular cell adhesion molecule-1 (VCAM-1) and intercellular adhesion molecule-1 (ICAM-1), which facilitate the adhesion and transmigration of leukocytes into the retinal microvasculature [164]. The infiltration of leukocytes further amplifies the inflammatory response by releasing MMPs, which degrade the ECM and disrupt the BRB [165]. In DR, hyperglycemia-induced oxidative stress and AGEs also upregulate VEGF, promoting the formation of fragile, leaky blood vessels that contribute to retinal edema and hemorrhage. This neovascularization worsens retinal ischemia and inflammation [166]. Moreover, the chronic inflammatory environment leads to pericyte apoptosis and subsequent capillary dropout, exacerbating the progression of DR [167] (Fig. 5).

## 5. Treatment Approaches Targeting Vascular Inflammaging

### 5.1 Biological and Immunosuppressive Therapies

Canakinumab, a human monoclonal antibody that selectively inhibits IL-1β, is a promising therapeutic agent for reducing systemic inflammation, particularly in the context of inflammaging. IL-1β plays a central role in activating the inflammasome, a key contributor to the chronic inflammation observed in aging and vascular diseases. By neutralizing IL-1β, canakinumab reduces the inflammatory burden and mitigates its vascular consequences [168]. The Canakinumab Anti-inflammatory Thrombosis Outcomes Study (CANTOS), a landmark phase III clinical trial, evaluated the effects of canakinumab in over 10,000 patients with a history of MI and elevated CRP levels [168]. Participants were randomized to receive subcutaneous doses of 50 mg, 150 mg, 300 mg, or a placebo every three months, with the primary endpoint being a composite of non-fatal MI, non-fatal stroke, or cardiovascular death. The trial revealed that canakinumab significantly reduced the incidence of the primary endpoint at the 150 mg dose compared to placebo [168]. Furthermore, a marked reduction in CRP levels confirmed the drug's anti-inflammatory efficacy. However, the study showed no significant reduction in all-cause mortality, and there was an increased risk of fatal infections, a notable adverse effect of IL-1β inhibition. The success of canakinumab in reducing cardiovascular events underscores its potential for treating vascular inflammaging by targeting IL-1β. However, the absence of an all-cause mortality benefit suggests that IL-1β inhibition may only address specific inflammatory pathways relevant to cardiovascular disease, leaving other pathways active. The increased infection risk highlights the need for careful patient selection and monitoring during treatment. [169]. While the CANTOS trial provided compelling evidence for the cardiovascular benefits of canakinumab, it also raised questions about its long-term efficacy and safety. For example, while IL-1β blockade reduces CRP, other inflammatory markers may remain elevated, contributing to ongoing inflammation in some individuals. Further research is needed to better understand the long-term effects of IL-1β inhibition, particularly in patients with comorbid conditions like diabetes or renal insufficiency, which increase the risk of infections. Additionally, combination therapies that could reduce infection risk, such as antimicrobial prophylaxis or immune response modulators, warrant exploration. Finally, there is a gap in understanding how IL-1β inhibition affects other inflammatory pathways, such as the NLRP3 inflammasome, NF-κB, or TNF-α signaling, which remain active despite IL-1β blockade. Further studies are necessary to explore the broader impacts of IL-1β inhibition on vascular inflammation and to optimize therapeutic strategies [168, 169].

Anakinra, an IL-1 receptor antagonist (IL-1Ra), blocks the activity of IL-1, a key pro-inflammatory cytokine involved in vascular inflammaging. By inhibiting IL-1, anakinra reduces systemic inflammation and mitigates its harmful effects on vascular tissues [170]. The MRC-ILA Heart trial evaluated the efficacy of anakinra in patients with acute myocardial infarction (AMI). Participants received daily subcutaneous injections of anakinra or placebo for 14 days, with the primary endpoint being the change in high-sensitivity C-reactive protein (hs-CRP) levels, a marker of systemic inflammation. Results demonstrated that anakinra significantly reduced hs-CRP levels compared to placebo, highlighting its potential anti-inflammatory effects [171]. Another key study, the Virginia Commonwealth University Anakinra Remodeling Trial (VCUART), focused on patients with heart failure. In this trial, participants were randomized to receive either anakinra or placebo for 14 days, with the primary endpoint being the change in left ventricular ejection fraction (LVEF). Anakinra significantly improved LVEF and reduced inflammatory markers, suggesting potential benefits in managing heart failure [172]. While anakinra has shown efficacy in reducing inflammation, its long-term safety profile and the duration of its anti-inflammatory effects require further investigation. Both the MRC-ILA Heart and VCUART trials evaluated only short-term outcomes (14 days), leaving critical gaps regarding the sustained impact of anakinra on vascular health and its long-term clinical benefits. Moreover, there is limited research on its effects in specific patient subgroups, such as those with comorbidities like diabetes or chronic kidney disease, who may exhibit altered or more pronounced inflammatory responses. Though reductions in inflammatory markers such as hs-CRP were observed in both trials, the long-term cardiovascular benefits of anakinra remain uncertain. Other studies have reported variable outcomes regarding mortality and hospitalization rates, suggesting that IL-1 inhibition alone may not sufficiently address the complex inflammatory mechanisms underlying cardiovascular diseases. Additionally, the broader effects of anakinra on other inflammatory pathways, including those involving cytokines like IL-6 or TNF-α, have yet to be fully elucidated. Future studies should aim to assess not only the long-term efficacy of anakinra but also potential risks, such as immunosuppression and increased susceptibility to infections, especially in elderly patients or those with compromised immune systems. Investigating the interplay between IL-1 and other inflammatory mediators will be crucial for optimizing therapeutic strategies and understanding how anakinra may fit into a broader treatment landscape for vascular inflammaging [171, 172].

Colchicine, traditionally used to treat gout, has emerged as a promising anti-inflammatory agent for vascular diseases linked to inflammaging. It exerts its effects by disrupting microtubule polymerization, thereby inhibiting NF-κB signaling and the assembly of the NLRP3 inflammasome. This leads to the reduced release of pro-inflammatory cytokines, including IL-1β and TNF-α, which are central to chronic inflammatory processes [173]. The Colchicine Cardiovascular Outcomes Trial (COLCOT), which included 4,745 patients with a recent MI, explored the efficacy of colchicine in preventing cardiovascular events. Participants were randomized to receive 0.5 mg of colchicine daily or placebo, with the primary composite endpoint being cardiovascular death, resuscitated cardiac arrest, MI, stroke, or urgent hospitalization for angina requiring revascularization. The trial demonstrated a significant reduction in the primary endpoint in the colchicine group, primarily driven by decreases in stroke and urgent revascularization events [174]. The Low-Dose Colchicine (LoDoCo) trial further investigated colchicine’s long-term safety and efficacy in patients with stable coronary artery disease. Involving 532 patients, this trial compared daily colchicine at 0.5 mg to no colchicine. The primary outcome, a composite of acute coronary syndrome, out-of-hospital cardiac arrest, or non-cardioembolic ischemic stroke, was significantly lower in the colchicine group, highlighting its protective effect in chronic coronary artery disease [175]. Building on the LoDoCo trial, the LoDoCo2 trial was a larger, multicenter study involving over 5,000 patients with stable CAD. Participants were randomized to receive either 0.5 mg of colchicine daily or placebo, with the primary endpoint being a composite of cardiovascular death, MI, ischemic stroke, or ischemia-driven coronary revascularization. The findings corroborated earlier results, showing a significant reduction in cardiovascular events in the colchicine group [176]. The success of colchicine in reducing cardiovascular events in both acute and chronic settings underscores its potential as a therapeutic option for managing vascular inflammaging. Its low cost and established safety profile further enhance its attractiveness in this context. However, while its anti-inflammatory effects on the NLRP3 inflammasome and NF-κB pathways are well-documented, the precise impact of colchicine on different inflammatory pathways during various stages of cardiovascular disease requires further study. The efficacy of the drug may vary depending on individual inflammatory profiles, comorbidities, and genetic factors. Biomarkers such as CRP or IL-1β could potentially be used to identify patients most likely to benefit from colchicine therapy. Additionally, the long-term safety of colchicine remains a concern, particularly its effects on gastrointestinal, renal, and hepatic functions. Although short-term safety was demonstrated in the trials, extended use in older populations, who are more susceptible to adverse effects, needs detailed evaluation. Furthermore, the drug-drug interactions of colchicine, especially with medications commonly used in cardiovascular disease management such as statins and anticoagulants, have not been thoroughly studied in clinical practice. Such interactions could influence the benefit-risk profile of colchicine, particularly in elderly patients or those undergoing polypharmacy treatment [174-176].

Proprotein convertase subtilisin/kexin type 9 (PCSK9) inhibitors, such as alirocumab and evolocumab, are monoclonal antibodies designed to target and inhibit PCSK9, a protein that degrades low-density lipoprotein receptors (LDLR) on hepatocytes. By inhibiting PCSK9, these drugs increase the availability of LDLR, enhancing the clearance of low-density lipoprotein cholesterol (LDL-C) from the bloodstream. Elevated LDL-C is a well-established risk factor for vascular inflammation and atherosclerosis, making PCSK9 inhibitors highly effective in reducing cardiovascular risk [177]. The FOURIER trial evaluated evolocumab in 27,564 patients with clinically evident atherosclerotic cardiovascular disease, all receiving statins. Participants were randomized to receive either evolocumab or placebo, with the primary composite endpoint being cardiovascular death, MI, stroke, hospitalization for unstable angina, or coronary revascularization. The results demonstrated that evolocumab significantly reduced the incidence of the primary endpoint, particularly by lowering the risk of MI, stroke, and coronary revascularization [178]. The ODYSSEY OUTCOMES trial assessed alirocumab in 18,924 patients who had recently experienced an acute coronary syndrome event. Participants were randomized to receive either alirocumab or placebo, with the primary endpoint being a composite of coronary heart disease death, non-fatal MI, fatal or non-fatal ischemic stroke, or unstable angina requiring hospitalization. Alirocumab significantly reduced the incidence of the primary endpoint, driven mainly by reductions in MI and ischemic stroke [179]. PCSK9 inhibitors have established efficacy in significantly reducing LDL-C levels and lowering the risk of subsequent cardiovascular events. Their potential role in addressing vascular inflammaging stems from their ability to reduce atherogenic lipoproteins and associated inflammation [180]. However, gaps remain in understanding their long-term effects on vascular inflammation and aging. For example, there is limited evidence on how PCSK9 inhibitors affect broader inflammatory pathways, particularly those involving pro-inflammatory cytokines such as IL-6, TNF-α, and IL-1β, which are key drivers of inflammaging. Given that PCSK9 inhibitors primarily target lipid metabolism rather than directly influencing inflammatory pathways, more research is needed to explore how these drugs interact with inflammatory mediators beyond LDL-C reduction. Furthermore, there is a need for additional studies focusing on the long-term use of PCSK9 inhibitors in elderly populations, particularly those with chronic inflammatory conditions. The elderly face an elevated risk of both atherosclerosis and inflammaging-related vascular dysfunction, and it is not yet clear whether PCSK9 inhibitors can fully address the complex interplay between lipid metabolism, oxidative stress, and inflammation in this demographic. Further investigation into the potential of PCSK9 inhibitors to modulate the immune response and reduce systemic inflammation is necessary to fully understand their role in treating vascular inflammaging [178, 179].

Inclacumab is a monoclonal antibody targeting P-selectin, a cell adhesion molecule crucial in leukocyte recruitment and platelet aggregation. By inhibiting P-selectin, inclacumab reduces leukocyte adhesion and transmigration, thereby decreasing inflammation and thrombosis within the vasculature [181]. The SELECT-ACS trial evaluated inclacumab in patients with non-ST-segment elevation myocardial infarction (NSTEMI). Participants received a single intravenous dose of inclacumab or placebo prior to undergoing percutaneous coronary intervention. The primary outcome was the reduction in myocardial damage, assessed by cardiac biomarkers. Inclacumab significantly reduced myocardial damage compared to placebo, suggesting a protective effect during percutaneous coronary intervention [182]. The SELECT-CABG trial focused on patients undergoing coronary artery bypass grafting (CABG). Participants were randomized to receive either inclacumab or placebo. However, the SELECT-CABG trial demonstrated limited practical benefit for inclacumab in improving outcomes after CABG surgery [183]. Despite the ability of inclacumab to reduce inflammation and thrombotic events, these mixed results highlight several areas of uncertainty. First, while inclacumab showed efficacy in reducing myocardial damage in percutaneous coronary intervention, this benefit did not extend to CABG patients. The differences in inflammatory and thrombotic processes between percutaneous coronary intervention and CABG may explain these discrepancies, but further research is required to understand the underlying mechanisms. Additionally, the long-term outcomes following inclacumab treatment remain largely unexplored. While the reduction in myocardial damage is promising, studies investigating whether inclacumab can decrease the incidence of recurrent cardiovascular events, such as MI or heart failure, are necessary to fully assess its clinical value. Moreover, there is limited understanding of the effects of inclacumab on systemic inflammation beyond the acute phase of coronary intervention. Given the central role of chronic low-grade inflammation in vascular aging (inflammaging), future research should explore whether inclacumab could provide benefits in reducing inflammation and thrombosis in aging-related vascular diseases, such as atherosclerosis and peripheral artery disease. Finally, the potential for inclacumab to be integrated into personalized medicine approaches is an area worth further exploration. Biomarkers such as P-selectin levels, leukocyte counts, and platelet activity could guide patient selection, helping ensure that inclacumab is administered to those most likely to benefit. This approach could optimize its clinical use, particularly in high-risk populations [182, 183].

Statins primarily function by inhibiting HMG-CoA reductase, a key enzyme in the cholesterol biosynthesis pathway, leading to reduced cholesterol levels [184]. Beyond their lipid-lowering effects, statins exhibit significant anti-inflammatory properties. They inhibit NF-κB activation, thereby reducing the expression of pro-inflammatory cytokines such as TNF-α, IL-6, and CRP. Statins also promote the stabilization of atherosclerotic plaques by decreasing inflammatory cell infiltration and reducing NOX activity, which in turn lowers ROS levels, mitigating oxidative damage and inflammatory responses. Furthermore, statins enhance the bioavailability of NO by upregulating eNOS, which exerts vasodilatory, anti-inflammatory, and anti-thrombotic effects, while reducing leukocyte and platelet adhesion to the endothelium [184, 185]. The Pravastatin or Atorvastatin Evaluation and Infection Therapy (PROVE-IT) trial was a landmark study comparing intensive lipid-lowering therapy with atorvastatin (80 mg/day) to standard therapy with pravastatin (40 mg/day) in patients who had experienced an acute coronary syndrome [186]. This trial, which included 4,162 participants, used a composite primary endpoint that encompassed death from any cause, MI, documented unstable angina requiring hospitalization, revascularization, and stroke [186]. The results showed that atorvastatin significantly reduced the incidence of the primary endpoint compared to pravastatin. Specifically, the atorvastatin group experienced fewer major cardiovascular events, underscoring the benefits of intensive lipid-lowering therapy. In addition, inflammatory markers such as CRP were significantly reduced in the atorvastatin group, suggesting a stronger anti-inflammatory effect, which appeared to be particularly beneficial for patients at high risk of recurrent cardiovascular events [186]. The Justification for the Use of Statins in Prevention: an Intervention Trial Evaluating Rosuvastatin (JUPITER) trial evaluated rosuvastatin in individuals with elevated CRP levels but normal LDL cholesterol levels [187]. This study involved 17,802 participants who were randomized to receive either 20 mg of rosuvastatin daily or a placebo. The primary endpoint included MI, stroke, arterial revascularization, hospitalization for unstable angina, or death from cardiovascular causes [187]. The trial was terminated early due to the significant benefits observed in the rosuvastatin group. Results demonstrated a 44% reduction in the primary endpoint, with substantial decreases in both LDL cholesterol and CRP levels. These findings strongly supported the anti-inflammatory benefits of rosuvastatin, independent of its lipid-lowering effects [187]. The JUPITER trial provided evidence that rosuvastatin is effective in reducing cardiovascular events in individuals with elevated inflammation markers, even when cholesterol levels are not markedly elevated. This broadened the scope of statin therapy to include primary prevention in patients with high CRP, emphasizing the critical role of inflammation in cardiovascular risk [187]. Despite the robust findings from the PROVE-IT and JUPITER trials, gaps and contradictions remain regarding the role of statins in inflammation. While statins consistently lower CRP levels, the clinical relevance of this reduction, particularly in primary prevention, remains debated. In some populations, the reduction in CRP and other inflammatory markers does not always correlate with a decreased incidence of cardiovascular events, suggesting that other factors may influence the efficacy of statins in addressing inflammation-driven cardiovascular risk. Additionally, uncertainty persists as to whether the anti-inflammatory effects of statins are directly responsible for improved clinical outcomes or are secondary to their lipid-lowering actions. These complexities underscore the multifaceted nature of inflammaging and suggest that targeting multiple inflammatory pathways may be necessary to achieve optimal cardiovascular risk reduction. Future research should aim to identify the inflammatory markers most predictive of statin efficacy and explore whether adjunct therapies targeting additional inflammatory pathways could enhance the cardiovascular benefits of statins [186, 187].

Methotrexate, a well-established folate antagonist, is primarily used as an anti-proliferative agent in cancer therapy and as an immunosuppressant in autoimmune diseases such as rheumatoid arthritis [188]. Its anti-inflammatory effects are mediated through several mechanisms, including the inhibition of dihydrofolate reductase, which leads to decreased synthesis of thymidylate and purines, essential components for DNA replication and cell proliferation. Additionally, methotrexate promotes the release of adenosine, a potent anti-inflammatory mediator, and suppresses the production of pro-inflammatory cytokines, including IFN-γ [188]. The Cardiovascular Inflammation Reduction Trial (CIRT) aimed to evaluate the impact of low-dose methotrexate on cardiovascular outcomes in patients with stable atherosclerosis and coexisting type 2 diabetes or metabolic syndrome [189]. The trial enrolled 4,786 participants, who were randomly assigned to receive either low-dose methotrexate (15-20 mg weekly) or a placebo, alongside folic acid supplementation to counteract potential side effects. The primary endpoint was a composite of non-fatal MI, non-fatal stroke, or cardiovascular death. Throughout the trial, inflammatory markers such as high-sensitivity CRP (hs-CRP), IL-6, and TNF-α were monitored. However, the results of the CIRT trial revealed that low-dose methotrexate did not significantly reduce the incidence of the primary endpoint compared to placebo. Moreover, there was no significant reduction in levels of IL-6, CRP, or other inflammatory markers in the methotrexate group. These findings contrasted with earlier observational studies and smaller trials, which had suggested that methotrexate might confer cardiovascular benefits. The CIRT trial thus underscored the complexities of translating anti-inflammatory therapies from other contexts to cardiovascular disease [189]. One potential explanation for the discrepancy between the CIRT trial and prior research is that the anti-inflammatory effects of methotrexate may be more effective in conditions where inflammation is directly linked to autoimmune mechanisms, as in rheumatoid arthritis. In contrast, in atherosclerosis, the inflammatory pathways may be more nuanced or involve different mediators. Additionally, the patient population in CIRT—characterized by inflammation driven by metabolic factors such as hyperglycemia and insulin resistance—may not respond to methotrexate in the same way as patients with autoimmune diseases. These metabolic factors may involve inflammatory pathways that are less responsive to methotrexate. Further research is needed to elucidate why methotrexate failed to show cardiovascular benefits in the CIRT trial despite its known anti-inflammatory properties. Key gaps include the need for a better understanding of the distinct inflammatory pathways involved in cardiovascular disease compared to autoimmune disorders. Moreover, it remains unclear whether higher doses of methotrexate or combination therapies with other anti-inflammatory agents could produce more substantial effects on cardiovascular outcomes. The failure to reduce inflammatory markers such as IL-6 and CRP in the CIRT trial suggests that the inflammatory mechanisms driving cardiovascular disease may be more complex than originally anticipated, and methotrexate may not effectively target these pathways in this context [189].

Darapladib is a selective inhibitor of lipoprotein-associated phospholipase A2 (Lp-PLA2), an enzyme involved in the inflammatory processes driving atherosclerosis [190]. Lp-PLA2 hydrolyzes oxidized phospholipids in low-density lipoprotein (LDL) particles, generating pro-inflammatory and pro-atherogenic byproducts such as lysophosphatidylcholine and oxidized nonesterified fatty acids. By inhibiting Lp-PLA2, darapladib aims to decrease vascular inflammation and slow the progression of atherosclerotic plaques [190]. The Stabilization of Atherosclerotic Plaque by Initiation of Darapladib Therapy (STABILITY) trial was conducted to assess whether darapladib could reduce cardiovascular events in patients with stable coronary heart disease [191]. The trial included 15,828 participants, who were randomized to receive either darapladib (160 mg daily) or placebo alongside standard care. The primary endpoint was a composite of major adverse cardiovascular events, including cardiovascular death, MI, or stroke [191]. The results of the STABILITY trial revealed that darapladib did not significantly reduce the primary endpoint of major adverse cardiovascular events compared to placebo. However, secondary analyses suggested a potential benefit in reducing the risk of major coronary events [191]. These findings indicate that darapladib may not be effective as a broad therapeutic option for all patients with coronary artery disease, underscoring the complexity of translating anti-inflammatory strategies into tangible clinical benefits for cardiovascular disease. While Lp-PLA2 is clearly involved in promoting inflammation and plaque instability, the failure of darapladib to significantly lower the risk of major adverse cardiovascular events raises questions about which specific inflammatory pathways should be targeted in atherosclerosis. One possibility is that inhibiting Lp-PLA2 alone may not sufficiently address the multifactorial progression of atherosclerotic disease, which includes not only inflammation but also lipid metabolism, endothelial dysfunction, and thrombosis. However, the potential role of darapladib in plaque stabilization and reducing the risk of major coronary events, as indicated by secondary analyses, suggests that it may still hold value in targeted patient subgroups. For example, individuals with high-risk plaque features, such as large necrotic cores, thin fibrous caps, or a history of recurrent coronary events, may benefit more from therapies specifically designed to stabilize vulnerable plaques [191].

Varespladib is an inhibitor of secretory phospholipase A2 (sPLA2), an enzyme involved in the inflammatory cascade that contributes to atherosclerosis. sPLA2 hydrolyzes phospholipids in cell membranes, releasing arachidonic acid, a precursor to pro-inflammatory eicosanoids. By inhibiting sPLA2, varespladib aims to reduce the production of inflammatory mediators and stabilize atherosclerotic plaques [192]. The Vascular Inflammation Suppression to Treat Acute Coronary Syndrome for 16 Weeks (VISTA-16) trial was designed to evaluate the efficacy of varespladib in reducing cardiovascular events in patients with acute coronary syndrome [193]. The trial involved 5,145 patients who were randomized to receive either varespladib (500 mg daily) or placebo, alongside standard acute coronary syndrome therapy. The primary endpoint was a composite of cardiovascular death, MI, stroke, or unstable angina requiring hospitalization [193]. However, subsequent analyses indicated that varespladib did not significantly reduce inflammation or improve lipid profiles, and safety concerns arose, particularly regarding an increased risk of MI [193]. The failure of varespladib to lower cardiovascular events in the VISTA-16 trial, along with these safety concerns, underscores the difficulties in translating anti-inflammatory therapies into effective treatments for cardiovascular disease. While sPLA2 plays a role in both the release of pro-inflammatory mediators and lipid metabolism, inhibiting it may disrupt protective mechanisms, potentially increasing the risk of plaque rupture and MI. Further research is needed to better understand the off-target effects of sPLA2 inhibition, which might alter the balance between pro- and anti-inflammatory lipid mediators, thereby promoting plaque destabilization rather than stabilization. Future studies should focus on identifying the full range of lipid metabolites influenced by sPLA2 inhibition and their effects on plaque biology and cardiovascular outcomes. Additionally, the inflammatory processes driving atherosclerosis in ACS may be too complex to be addressed by sPLA2 inhibition alone. The failure to reduce inflammatory markers like hs-CRP and the lack of improvement in lipid profiles suggest that sPLA2 may not be a central player in acute atherothrombosis, or at least not one that can be easily modulated by pharmacological inhibition. This highlights the need for further research investigation into the specific inflammatory pathways involved in ACS and the exploration of alternative therapeutic targets to more effectively reduce cardiovascular risk [192, 193].

## 5.2 Antioxidant Therapies

### 5.2.1 Synthetic Antioxidants

Coenzyme Q10 (CoQ10), also known as ubiquinone, is a lipid-soluble antioxidant integral to mitochondrial electron transport and energy production. CoQ10 exists in both its reduced (ubiquinol) and oxidized (ubiquinone) forms, enabling it to effectively scavenge ROS and regenerate other antioxidants. Through its ability to reduce oxidative stress, CoQ10 can improve endothelial function, inhibit lipid peroxidation, and modulate the expression of pro-inflammatory cytokines [194]. A meta-analysis of randomized controlled trials in hypertensive patients demonstrated that CoQ10 supplementation significantly reduced both systolic and diastolic blood pressure, suggesting that its antioxidant properties improve endothelial function and reduce vascular resistance, contributing to enhanced blood pressure control [195]. In a double-blind, placebo-controlled trial involving patients with coronary artery disease, CoQ10 supplementation led to significant improvements in endothelial function, as measured by flow-mediated dilation (FMD), and reductions in oxidative stress markers such as 8-isoprostane [196]. Additionally, the Q-SYMBIO study, which investigated the long-term effects of CoQ10 in patients with chronic heart failure, found that CoQ10 supplementation significantly reduced major adverse cardiovascular events and improved overall survival rates, highlighting its potential in severe cardiovascular conditions [197]. The ability of CoQ10 to enhance mitochondrial function and reduce oxidative stress positions as a valuable therapeutic agent in addressing vascular inflammaging. Its demonstrated efficacy in improving endothelial function and reducing cardiovascular events underscores its potential in the treatment of various vascular diseases [198]. However, despite the encouraging evidence, not all studies have consistently shown reductions in blood pressure, oxidative stress, or inflammation with CoQ10 supplementation. These discrepancies may be due to variations in study designs, heterogeneity in patient populations, and differences in CoQ10 dosing regimens. One key area for future investigation is the bioavailability of CoQ10. Due to its poor absorption in oral form, factors such as age, gastrointestinal health, and co-administration with lipids can significantly influence its absorption. Future research should focus on optimizing CoQ10 formulations and dosing strategies to improve its bioavailability, which could enhance its therapeutic efficacy. Additionally, further studies are needed to clarify the specific mechanisms by which CoQ10 exerts its cardiovascular benefits. Although CoQ10 is known to reduce oxidative stress and improve mitochondrial function, its direct effects on pro-inflammatory cytokine expression and immune cell function in the context of vascular disease remain incompletely understood. Exploring these mechanisms in more detail could help refine the use of CoQ10 in personalized medicine approaches for patients with vascular inflammaging [196, 197].

N-acetylcysteine (NAC) is a precursor to L-cysteine, which is vital for the synthesis of glutathione, one of the body's major intracellular antioxidants. NAC enhances glutathione levels, directly scavenges ROS, and modulates the redox state of cells, thereby reducing oxidative stress [199]. Additionally, NAC can inhibit the activation of the NF-κB pathway, a key regulator of inflammation. Moreover, NAC promotes the polarization of macrophages toward the anti-inflammatory M2 phenotype, further mitigating inflammation [200]. Several clinical studies have investigated the efficacy of NAC in reducing oxidative stress and inflammation in vascular diseases. In a randomized controlled trial involving patients with chronic kidney disease, NAC supplementation significantly reduced markers of oxidative stress, such as MDA, and improved endothelial function, as evidenced by enhanced FMD [201]. Another study examining the effects of NAC on aortic fibrosis in aging mice found that NAC administration promoted M2 macrophage polarization, which is associated with anti-inflammatory and tissue repair processes. The study also demonstrated that NAC treatment reduced collagen deposition and improved aortic stiffness, highlighting its potential to reverse age-related vascular fibrosis [202]. NAC holds promise as an antioxidant therapy for managing vascular inflammaging. Its ability to reduce oxidative stress, inhibit pro-inflammatory pathways, and promote tissue repair positions it as a potential therapeutic agent for conditions such as atherosclerosis, hypertension, and age-related vascular stiffness. Future research may further elucidate its role in targeting the complex mechanisms of vascular inflammation and aging.

Vitamin E (α-tocopherol) and vitamin C (ascorbic acid) are potent antioxidants that neutralize ROS and protect cellular components from oxidative damage [203]. Vitamin E, a lipid-soluble antioxidant, is incorporated into cell membranes where it prevents lipid peroxidation. Vitamin C, a water-soluble antioxidant, regenerates oxidized vitamin E and directly scavenges ROS, supporting cellular defense against oxidative stress [204]. Despite their antioxidant properties, large clinical trials have questioned the efficacy of vitamin E and vitamin C supplementation in reducing cardiovascular risk. The Heart Outcomes Prevention Evaluation (HOPE) and HOPE-TOO trials, which assessed vitamin E supplementation (400 IU/day) in patients at high risk of cardiovascular events, found no significant reduction in major cardiovascular events. In fact, in some cases, an increased risk of heart failure was observed [205]. Similarly, the Women's Antioxidant Cardiovascular Study (WACS), which evaluated vitamin C (500 mg/day) and vitamin E (600 IU every other day) in 8,171 women at high risk for cardiovascular disease, also showed no significant reduction in the incidence of major cardiovascular events with either antioxidant [206]. While the antioxidant activity of vitamins E and C is well-established in vitro, their clinical efficacy in reducing cardiovascular events through supplementation remains inconclusive. These findings suggest that antioxidant supplementation may not translate into meaningful cardiovascular benefits, highlighting the complexity of oxidative stress in disease progression and the need for a more nuanced understanding of antioxidant therapy.

### 5.2.2 Natural Compounds

Resveratrol, a polyphenolic compound found in red wine, grapes, and berries, is known for its anti-inflammatory and antioxidant properties, primarily mediated through the activation of SIRT1. By activating SIRT1, resveratrol deacetylates various transcription factors, leading to a reduction in pro-inflammatory cytokine expression and oxidative stress, which helps protect endothelial cells from damage [207]. Resveratrol also enhances mitochondrial function and biogenesis, which contributes to improved cellular health and mitigates inflammaging [208]. Several clinical trials have investigated the effects of resveratrol supplementation on cardiovascular health, particularly in patients with metabolic syndrome. These studies reported significant improvements in endothelial function, as measured by FMD, along with reductions in oxidative stress and inflammatory markers such as CRP and IL-6 [209]. Additionally, resveratrol supplementation was associated with a significant slowing in carotid intima-media thickness (CIMT) progression compared to placebo [210]. Reductions in LDL cholesterol and inflammatory biomarkers further underscore the anti-atherosclerotic potential of the compound [211].

Quercetin, a flavonoid abundant in fruits, vegetables, and grains, exhibits powerful antioxidant and anti-inflammatory properties. Its anti-inflammatory effects are largely driven by the inhibition of the NF-κB pathway, which reduces the production of pro-inflammatory cytokines such as TNF-α, IL-1β, and IL-6 [212]. In addition, quercetin activates the Nrf2 pathway, leading to the upregulation of antioxidant enzymes like SOD and catalase, thereby strengthening the body’s antioxidant defenses [213]. Clinical trials on quercetin supplementation have shown improved endothelial function and reduced oxidative stress and inflammation, with notable reductions in CRP and TNF-α levels [214]. In other studies, quercetin supplementation in individuals with early-stage atherosclerosis led to a significant reduction in CIMT progression, improvements in lipid profiles, and a decrease in inflammatory markers, highlighting its anti-atherosclerotic potential [215].

Icariin, a flavonoid glycoside derived from the epimedium plant (commonly known as horny goat weed), has demonstrated considerable anti-inflammatory and antioxidant activity. Icariin inhibits the NF-κB signaling pathway, which helps lower the production of pro-inflammatory cytokines. It also activates the Nrf2 pathway, boosting antioxidant enzyme expression and protecting endothelial cells from oxidative damage [216]. Furthermore, icariin promotes the activation of the PI3K/Akt/eNOS pathway, leading to increased NO production and improved endothelial function. Animal studies on atherosclerosis have shown that icariin significantly enhances endothelial function, as evidenced by elevated eNOS expression and NO production, while also reducing oxidative stress markers and pro-inflammatory cytokines [217, 218]. Clinical trials on hyperlipidemic patients reported reductions in LDL cholesterol, triglycerides, and CRP levels with icariin treatment, alongside improved endothelial function, indicating potential anti-atherosclerotic benefits [219].

Curcumin, the active compound in turmeric, is well-known for its potent anti-inflammatory and antioxidant effects. Curcumin exerts its anti-inflammatory effects mainly by inhibiting NF-κB, thereby reducing the expression of pro-inflammatory cytokines [220]. In addition, curcumin boosts the activity of antioxidant enzymes like SOD and catalase, helping to reduce oxidative stress. Studies have also shown the ability of curcumin to lower LDL cholesterol and inflammatory biomarkers, suggesting its therapeutic potential for managing atherosclerosis and inflammation [221]. Ginkgo biloba extract (GBE) inhibits platelet-activating factor (PAF) and modulates NO production, which plays a crucial role in maintaining endothelial function and reducing vascular inflammation [222]. The flavonoids in GBE enhance eNOS activity, increasing NO bioavailability and improving vascular health [223]. Additionally, GBE inhibits the activation of NF-κB, leading to a decrease in pro-inflammatory cytokine expression. These effects position GBE as a potential treatment for atherosclerosis and other age-related vascular diseases [224] (Table 1).

**Table 1:** Clinical trials of anti-inflammatory therapy in vascular inflammatory diseases.

Trial/PMID	Medication	Target	Condition	Patients (n)	Duration	Results/Outcomes
**PROVE-IT/15007110**	Atorvastatin Pravastatin	Cholesterol	ACS	4,162	24 months	High-dose atorvastatin reduced levels of CRP and provided significantly better protection against death or major cardiovascular events compared to moderate-dose pravastatin.
**JUPITER/18997196**	Rosuvastatin	Cholesterol	normal levels of LDL-C but elevated levels of hs-CRP	17,802	1.9 years	Significantly reduced the levels of hs-CRP and the incidence of major cardiovascular events
**LoDoCo/23265346**	Colchicine	Microtubule assembly	SCD	532	3 years	Effectively reduced the cardiovascular events in patients with SCD
**SELECT-ACS/23500230**	Inclacumab	P-selectin	NSTEMI	544	4 months	Significantly reduced levels of troponin I and CK-MB, thereby reducing myocardial damage after PCI in patients with NSTEMI.
**STABILITY/24678955**	Darapladib	Lp-PLA2	SCHD	15,828	3.7 years	No reduction of fatal and non-fatal cardiovascular events in patients with SCHD.
**VISTA-16/24247616**	Varespladib	sPLA2	ACS	5,145	16 weeks	No reduction of the risk of recurrent cardiovascular events and an increased risk of MI.
**Q-SYMBIO/25282031**	CoQ10	Mitochondrial Electron Transport	HF	420	2 years	Improvement of the symptomatic, and reduce of major adverse cardiovascular events
**MRC-ILA Heart/25079365**	Anakinra	IL-1R	NSTE-ACS	182	12 months	Reduction in inflammatory markers two weeks following NSTE-ACS
**SELECT-CABG/26796402**	Inclacumab	P-selectin	undergoing CABG surgery	384	1 year	Inability to reduce saphenous vein graft disease after CABG surgery.
**CANTOS/28845751**	Canakinumab	IL-1β	MI	10,061	3.7 years	Significantly lowered rate of recurrent cardiovascular events
**FOURIER/28304224**	PCSK9 inhibitors	PCSK9	ASCVD	27,564	2.2 years	Effectively reduced the incidence of major adverse cardiovascular events
**ODYSSEY OUTCOMES/30403574**	PCSK9 inhibitors	PCSK9	ACS	18,924	2.8years	Effectively reduced cardiovascular risk in high-risk patients with recent ACS
**COLCOT/31733140**	Colchicine	Microtubule assembly	MI	4745	22.6 months	Effectively reduced the risk of ischemic cardiovascular events in patients with a recent MI and has a favorable safety profile
**CIRT/30415610**	Methotrexate	Purinergic signalling	MI or MVD with T2DM or metabolic syndrome.	4,786	2.3 years	No reduction of inflammatory markers and cardiovascular events in patients with stable atherosclerosis
**LoDoCo2/32865380**	Colchicine	Microtubule assembly	CCD	5522	28.6 months	Significantly reduced the risk of cardiovascular events in patients with chronic coronary disease
**VCUART/34617567**	Anakinra	IL-1R	STEMI	139	12 months	Notable decrease in systemic inflammatory response with a lowered occurrence of HF and hospitalizations for HF.

Abbreviations: ACS, acute coronary syndrome; ASCVD, atherosclerotic cardiovascular disease; CABG, coronary artery bypass graft; CCD, chronic coronary disease; CK-MB, creatine kinase-MB; CoQ10, coenzyme Q10; CRP, C-reactive protein; HF, heart failure; hs-CRP, high-sensitivity C-reactive protein; IL-1β, interleukin-1β; IL-1R, interleukin-1 receptor; LDL-C, low-density lipoprotein cholesterol; Lp-PLA2, lipoprotein-associated phospholipase A2; MI, myocardial infarction; MVD, multivessel coronary disease; NSTE-ACS, non-ST elevation acute coronary syndrome; NSTEMI, non-ST-segment elevation myocardial infarction; PCI, percutaneous coronary intervention; PCSK9, proprotein convertase subtilisin/kexin type 9; SCD, stable coronary disease; SCHD, stable coronary heart disease; sPLA2, secretory phospholipase A2; STEMI, ST-elevation myocardial infarction; T2DM, type 2 diabetes mellitus.

### 5.3 Renin-Angiotensin-Aldosterone System Modulators

Angiotensin-converting enzyme (ACE) inhibitors (ACEIs) and ARBs are pivotal therapeutic agents for managing vascular inflammaging. ACEIs work by inhibiting the conversion of angiotensin I to angiotensin II, a potent vasoconstrictor that promotes inflammation, while ARBs prevent angiotensin II from binding to its receptors, mitigating its detrimental effects on the vasculature. By reducing angiotensin II levels, both classes of drugs decrease vascular inflammation and oxidative stress, while also improving endothelial function through enhanced NO bioavailability [225]. Research has shown that ACEIs and ARBs suppress the expression of pro-inflammatory cytokines and adhesion molecules, both of which play key roles in vascular inflammation and aging. Inhibition of angiotensin II has also been linked to reduced superoxide production, which helps preserve endothelial function [226]. Moreover, the preservation of endothelial-derived hyperpolarizing factor (EDHF) function in the presence of ACEI/ARB therapy supports microvascular homeostasis, particularly when NO-mediated vasodilation is compromised [100]. These therapies have also been shown to improve vascular repair and regeneration by enhancing the function of endothelial progenitor cells, thereby promoting vascular healing post-injury. Additionally, ACEIs and ARBs help mitigate the increased arginase activity often seen in endothelial dysfunction, further preserving vascular health [227]. In clinical settings, the use of ACEIs and ARBs has been associated with reductions in major adverse cardiovascular events and improved survival rates in patients with chronic heart conditions. Their broad therapeutic benefits, including the improvement of endothelial function and reduction of cardiovascular events, underscore their significance in managing severe cardiovascular diseases and combating vascular inflammaging. Their role in reducing inflammation and enhancing vascular health makes them valuable tools in treating a range of vascular diseases [228].

## 6. Conclusions and Future Directions

In conclusion, the mechanisms driving vascular inflammaging highlight a crucial link between chronic inflammation and vascular pathology, underscoring the urgent need for innovative therapeutic strategies and research pathways. It is essential to shift our research focus toward advanced biomedical methodologies, including the creation of predictive aging models that accurately simulate vascular inflammaging. Such models could provide a solid foundation for generating reliable data to better understand complex inflammatory mechanisms. Additionally, a significant gap remains in the identification and targeting of the molecular and cellular mediators of inflammaging. Future therapeutic strategies should prioritize the design and development of targeted drugs aimed at modulating key inflammatory pathways central to the aging vascular system. However, translating these targets into clinical applications faces challenges due to technical hurdles in drug development, such as the need for precise drug delivery systems and the minimization of off-target effects. Addressing these challenges will require a coordinated effort to deepen our understanding of inflammaging at molecular, cellular, and systemic levels. This progress will pave the way for interventions that can alleviate the vascular complications associated with aging.
